# The effect of atorvastatin combined with azithromycin on lung function and right heart function in COPD patients with concomitant pulmonary hypertension

**DOI:** 10.3389/fmed.2025.1667773

**Published:** 2026-01-29

**Authors:** Weiwei Que, Saiyu Li, Jin Li, Youtang Li, Zhiyi Ma

**Affiliations:** 1 Pulmonary and Critical Care Medicine, The First Hospital of Longyan Affiliated to Fujian Medical University, Longyan, China; 2 Cardiovascular Medicine, The First Hospital of Longyan Affiliated to Fujian Medical University, Longyan, China

**Keywords:** atorvastatin, azithromycin, lung function, right heart function, COPD, pulmonary hypertension

## Abstract

**Background:**

Chronic obstructive pulmonary disease (COPD) often coexists with pulmonary hypertension (PH), leading to exacerbated morbidity and mortality. Conventional treatments focus on bronchodilation, anti-inflammatory agents, and vasodilators, emphasizing the need for novel interventions. Atorvastatin and azithromycin have pleiotropic properties that may benefit COPD with concomitant PH. However, the combined impact of these medications on lung and right heart function in this patient population remains underexplored.

**Methods:**

A retrospective cohort study involving 229 stable COPD patients with concomitant PH assessed the effects of atorvastatin combined with azithromycin. Patients were divided into atorvastatin monotherapy and atorvastatin plus azithromycin combination therapy groups and underwent a 6-month treatment period. Blood testing, pulmonary function testing, and echocardiographic assessment of right heart function were conducted before and after treatment.

**Results:**

The combination therapy group exhibited significant improvements in blood gas indicators (mean PaO_2_ increase: 1.22 kPa, 95% CI: 0.82–1.62 kPa, *p* < 0.001), lung function (mean FEV1 increase: 0.18 L, 95% CI: 0.08–0.28 L, *p* < 0.001), right heart function (mean PAPs reduction: 8.0 mmHg, 95% CI: 7.0–9.0 mmHg, *p* < 0.001), respiratory distress, and daily living ability compared to the monotherapy group. Positive correlations were observed between improvements in pulmonary function and daily living ability. The combination therapy resulted in a significantly higher total effective rate (84.48% vs. 72.73%, *p* = 0.042) compared to the monotherapy group.

**Conclusion:**

This retrospective study suggests potential associations between combination therapy with atorvastatin and azithromycin and improvements in functional outcomes in COPD patients with concomitant PH. These findings warrant prospective validation in randomized controlled trials.

## 1 Introduction

Chronic obstructive pulmonary disease (COPD) is a debilitating respiratory condition characterized by progressive airflow limitation and persistent respiratory symptoms, often exacerbated by concomitant pulmonary hypertension (PH). The coexistence of COPD and PH presents a complex clinical scenario with significant implications for patient management and outcomes (1, 2). While COPD primarily affects the airways and lung parenchyma, PH involves elevated pulmonary artery pressure, leading to right heart dysfunction and increased morbidity and mortality (3–5). Both conditions impose a substantial burden on affected individuals and healthcare systems, warranting comprehensive therapeutic approaches (6–8).

The pathogenesis of COPD and PH is intricately interconnected, involving inflammatory processes, endothelial dysfunction, vascular remodeling, and impaired gas exchange. Management of COPD patients with concomitant PH represents a clinical challenge, requiring a multifaceted therapeutic strategy to address both pulmonary and cardiovascular manifestations (9–11). Conventional treatments typically focus on bronchodilation, anti-inflammatory agents, vasodilators, and oxygen therapy, but novel interventions that can effectively modulate the shared pathophysiological pathways remain needed (12, 13).

Accumulating evidence indicates that statins, especially atorvastatin, exert direct pulmonary-vascular benefits in COPD-PH. Atorvastatin has been demonstrated to exert anti-inflammatory effects on the pulmonary vasculature, improve endothelial function, and attenuate vascular remodeling (14–16). Studies have shown that atorvastatin can reduce pulmonary artery pressure and improve right ventricular function in patients with pulmonary hypertension. Similarly, azithromycin, a macrolide antibiotic with immunomodulatory properties, has been associated with reductions in airway inflammation and anti-remodeling effects in COPD (17). Randomized and cohort studies have reported that long-term azithromycin reduces COPD exacerbation frequency and improves FEV1, while small observational series show atorvastatin lowers PAPs in COPD-PH.

The potential synergistic effects of atorvastatin and azithromycin in modulating pulmonary and cardiovascular function in COPD with concomitant PH have not been extensively explored. Therefore, there is a compelling rationale to investigate the therapeutic potential of combining atorvastatin with azithromycin in addressing the multifactorial nature of COPD and PH.

## 2 Materials and methods

### 2.1 Study design

This retrospective cohort study included 229 stable COPD patients with concomitant PH admitted to our hospital from June 2020 to June 2023. The study collected patients’ demographic information, general data, routine blood tests, blood gas analysis, pulmonary function, echocardiographic right heart function parameters, adverse reaction symptoms, and scores from the Modified Medical Research Council Dyspnea Scale (MRC) and the Activities of Daily Living (ADL) scale.

Given the de-identified nature of the patient data used in this retrospective study, there was no potential for harm or impact on patient care. Therefore, the requirement for written informed consent was waived by the Institutional Review Board and Ethics Committee of First Hospital of Longyan Affiliated to Fujian Medical University (Approval No. FJLY-3255), in accordance with the Declaration of Helsinki and local regulatory guidelines for retrospective studies using de-identified data.

### 2.2 Inclusion and exclusion criteria

Inclusion criteria: (1) Age 40–80 years, no history of mental illness, normal cognitive function, and ability to cooperate with various treatments and examinations; (2) Symptomatic COPD (despite receiving maintenance inhalation therapy ≥2 times, COPD Assessment Test score ≥10 at screening) and forced expiratory volume in 1 second (FEV1) post-bronchodilator between 25% and 65% of predicted normal; (3) Pulmonary artery systolic pressure (PAPs) > 40 mmHg diagnosed as PH using echocardiography; (4) Class I–II heart function; (5) Patients who were informed and voluntarily agreed to participate in this study.

Exclusion criteria: (1) Unstable vital signs such as heart rate, body temperature, and blood pressure; (2) Severe cognitive impairment, visual or hearing impairments, mental illness history, inability to comply with treatment or examination, Mini-Mental State Examination (MMSE) score <24; (3) Active liver disease or elevated alanine aminotransferase due to other reasons, interstitial pneumonia, pulmonary tuberculosis, bronchogenic carcinoma, lung abscess, bronchiectasis, and other pulmonary diseases; (4) Patients with heart valve disease, congenital heart disease, requiring non-invasive positive pressure ventilation; (5) Patients with impaired liver or kidney function, recent use of corticosteroids, antibiotics, endothelin receptor antagonists, prostaglandin derivatives, or other vasodilators; (6) Known prolonged QTc interval (>480 ms) or concurrent use of QT-prolonging medications; (7) Known macrolide hypersensitivity or statin intolerance.

### 2.3 Grouping and treatment methods

Patients were divided into two groups based on the different treatment methods: the monotherapy group and the combination therapy group. In the monotherapy group (*n* = 113), patients were administered 20 mg of atorvastatin orally once daily at bedtime, with the option to adjust the dosage based on individual circumstances, not to exceed 80 mg per day. In the combination therapy group (*n* = 116), in addition to atorvastatin as described above, patients were given azithromycin 250 mg orally once daily (median prescribed dose = 250 mg qd, IQR 250–250 mg) for 6 months. Both groups of patients underwent a 6-month treatment period.

All participants continued standard-of-care inhaled therapy throughout the study (≥1 long-acting bronchodilator; 72% combined ICS/LABA). Exact baseline regimen is summarised in Supplementary Table S1.

Owing to local antimicrobial-stewardship policy at our institution, chronic macrolide prophylaxis is restricted to COPD patients with ≥3 exacerbations per year to minimize antimicrobial resistance. During the study window (June 2020 to June 2023), only 14 patients fulfilled both this criterion and our other inclusion criteria; all 14 were already receiving atorvastatin therapy. Therefore, an azithromycin-only cohort of adequate sample size could not be constituted. This represents a limitation in isolating the individual contributions of each medication. However, the combination group’s benefits exceeded those reported for azithromycin monotherapy in similar populations, suggesting at least additive effects.

Regarding antimicrobial stewardship and safety considerations, all patients prescribed azithromycin underwent baseline electrocardiogram (ECG) screening for QTc interval and were monitored at 1, 3, and 6 months with repeat ECG, liver enzyme testing (ALT, AST), and creatine kinase (CK) assessment. Patients were counseled about potential adverse effects and instructed to report symptoms such as palpitations, dizziness, abdominal pain, or muscle weakness. Treatment adherence was assessed through pill counts at each follow-up visit. One patient in the combination group discontinued azithromycin at 4 months due to gastrointestinal intolerance but was included in intention-to-treat analysis. We acknowledge that long-term macrolide use carries theoretical risks of antimicrobial resistance development, QT interval prolongation, and potential microbiome disruption; however, in this 6-month observational window, we did not systematically assess antimicrobial resistance patterns or perform comprehensive microbiome analyses. These remain important areas for future investigation.

### 2.4 Blood testing

Blood samples (5 mL) were obtained from the patients’ fasting venous blood before 8:00 a.m. for the following tests. The Beckman Coulter, Inc. DxH800 hematological analyzer (Brea, CA, USA) was used to measure red blood cells, white blood cells, neutrophils, lymphocytes, eosinophils, basophils, hemoglobin, and platelets. C-reactive protein levels were determined using the BECKMAN Synchron lx20 fully automatic biochemical analyzer (Beckman Coulter, Inc., Brea, CA, USA) through rate nephelometry. Whole blood anticoagulated with ethylenediaminetetraacetic acid (EDTA) was used for erythrocyte sedimentation rate (ESR) determination employing the TEST1 fully automatic ESR analyzer (ALIFAX, Inc., Italy). The supernatant obtained after centrifugation at 3,000 rpm for 5 min was used for the detection of procalcitonin (PCT), tumor necrosis factor alpha (TNF-α), interleukin-6 (IL-6), and interleukin-8 (IL-8) using enzyme-linked immunosorbent assay with the following reagent kits: PCT (ab221828, abcam, USA), TNF-α (ab181421, abcam, USA), IL-6 (ab178013, abcam, USA), and IL-8 (ab185986, abcam, USA). Arterial blood samples (2 mL) were collected at any time from the patients and the blood gas parameters were measured using the Beckman Coulter, Inc. blood gas analyzer (Brea, CA, USA). The arterial oxygen pressure (PaO_2_) and arterial carbon dioxide pressure (PaCO_2_) before and after treatment were statistically analyzed and compared between the two patient groups.

### 2.5 Pulmonary function testing

Lung function parameters, including FEV1, FVC, FEV1/FVC (%), DLCO, FEF50%, FEF75%, and MMEF, were measured using a comprehensive pulmonary function analyzer (Jaeger, Inc., Bodnegg, Germany) according to American Thoracic Society/European Respiratory Society guidelines. All tests were performed by trained respiratory technicians who were blinded to the treatment assignment. Quality control was maintained through regular calibration and adherence to standardized protocols. These parameters were measured before and after treatment in both patient groups, and the results were compared and statistically analyzed between the two groups.

### 2.6 Right heart function testing

Echocardiographic assessment of right heart function was performed using M-mode, two-dimensional, spectral Doppler, and tissue Doppler echocardiography with a Philips iE33 (USA) color Doppler ultrasound diagnostic device. Three probes were utilized: a convex array probe with a frequency range of 1.0–5.0 MHz, a linear array probe with a frequency range of 4.0–18 MHz, and a phased array probe with a frequency of 5 MHz.

All echocardiographic examinations were performed by two experienced cardiac sonographers (each with >10 years of experience in echocardiographic assessment of PH) who were blinded to patient treatment assignment and clinical outcomes. Each examination followed a standardized protocol as recommended by the American Society of Echocardiography and European Association of Cardiovascular Imaging guidelines. The following right heart function parameters were measured: right ventricular Tei index [calculated as (isovolumic contraction time + isovolumic relaxation time) / ejection time], right ventricular basal diameter measured in the apical four-chamber view (cm), right ventricular free wall thickness measured at end-diastole (cm), and PAPs (mmHg) estimated from tricuspid regurgitation peak velocity using the simplified Bernoulli equation plus estimated right atrial pressure based on inferior vena cava diameter and collapsibility.

To assess measurement reliability, 30 randomly selected echocardiograms (13% of total) were independently analyzed by both sonographers. Intra-observer agreement was assessed by having one sonographer repeat measurements on 30 echocardiograms after a 2-week interval. Inter-observer intraclass correlation coefficient (ICC) was 0.89 (95% CI: 0.81–0.94) for PAPs, and intra-observer ICC was 0.92 (95% CI: 0.85–0.96) for PAPs, indicating excellent reliability.

We acknowledge that echocardiographic estimation of PAPs, while validated against right heart catheterization in numerous studies and recommended as the primary non-invasive screening tool by international guidelines (ESC/ERS 2022), has inherent limitations. Right heart catheterization remains the gold standard for PH diagnosis and hemodynamic assessment. However, given the retrospective nature of this study and the invasive nature of right heart catheterization, echocardiography was deemed the most appropriate and feasible method for serial assessment of right heart function in this population. We selected the RV Tei index, basal diameter, free-wall thickness, and PAPs because these echo-derived metrics sensitively reflect afterload and have been recommended as pragmatic surrogates when right-heart catheterisation is not feasible. These parameters were statistically analyzed and compared between the two patient groups before and after treatment.

### 2.7 MRC score and ADL score

The Modified Medical Research Council (MRC) Dyspnea Scale was utilized to assess breathing difficulties in patients, with a score of 0 denoting the absence of shortness of breath during strenuous exercise, while 1 point signifies shortness of breath only during vigorous physical activity. A score of two indicates shortness of breath during stair or slope climbing, and a score of three denotes shortness of breath during walking compared to same-age peers on level ground. Finally, if a patient was unable to walk more than 90 meters without stopping due to shortness of breath or needed to stop for breath after walking a few minutes on level ground, they received a score of 4. The Cronbach’s alpha for the MRC was found to be 0.92 (18).

Measurement of the ADL score involved utilizing the Barthel Index to assess the patient’s daily activity ability, encompassing 10 distinct items (feeding, bathing, grooming, dressing, bowel control, bladder control, toilet use, transfers, mobility, and stairs). Each item was scored with higher points indicating greater independence, resulting in a maximum total score of 100 points. A higher score reflects stronger daily activity ability. The reliability of the ADL score was reported as 0.99 (19).

### 2.8 Efficacy evaluation

Clinical efficacy was evaluated using a three-category classification system based on objective functional and symptomatic improvements. “Significant Effect” was defined as: (1) improvement in FEV1 ≥ 200 mL or ≥12% from baseline, (2) reduction in PAPs ≥10 mmHg, (3) improvement in MRC score ≥1 point, (4) improvement in PaO2 ≥ 1.0 kPa, and (5) resolution or marked reduction of clinical symptoms (chronic cough, dyspnea, palpitations) with no recurrence within the 6-month observation period, with patients meeting at least three of these five criteria. “Effective” was defined as: meeting 1–2 of the above criteria with noticeable symptomatic improvement and occasional mild relapses post-treatment that did not require treatment intensification. “Ineffective” was defined as: meeting none of the above criteria, with no improvement in clinical symptoms and signs, or worsening of the condition requiring treatment modification or hospitalization.

The total effective rate was calculated using the formula: (significant + effective)/total number of cases × 100%. We acknowledge that this composite efficacy measure includes subjective components and may overestimate benefit compared to single objective endpoints; therefore, we also report individual objective outcomes (ΔFEV1, ΔPAPs, ΔPaO_2_) separately in the Results section to allow independent interpretation.

### 2.9 Statistical method

Continuous data were tested for normality using the Shapiro–Wilk test and assessed for homogeneity of variance using Levene’s test. Normally distributed data with homogeneous variance were presented as mean ± standard deviation (SD) and compared between groups using unpaired *t*-tests. Non-normally distributed data or data with heterogeneous variance were presented as median (interquartile range) and compared using Mann–Whitney U tests. Categorical data were expressed as frequency and percentage and compared using chi-square tests or Fisher’s exact test when appropriate.

For primary outcomes (blood gas, pulmonary function, and right heart function parameters), pre-post changes (*Δ*) were calculated as post-treatment value minus pre-treatment value, with negative *Δ* indicating improvement for parameters where lower values are better (e.g., PaCO_2_, PAPs, MRC score) and positive *Δ* indicating improvement for parameters where higher values are better (e.g., PaO_2_, FEV1, ADL score). Between-group comparisons of Δ values were performed using unpaired *t*-tests, and 95% confidence intervals (CI) were calculated for all mean differences.

Univariate and multivariable logistic regression analyses were performed to assess the association between treatment group and clinical efficacy outcome while adjusting for potential confounders including age, sex, BMI, smoking status, baseline exacerbation frequency, baseline FEV1, baseline PAPs, presence of hypertension, and diabetes mellitus. Odds ratios (OR) and 95% confidence intervals were calculated. Variables with *p* < 0.10 in univariate analysis were included in the multivariable model.

Missing values were observed in <5% of cases for any single variable and were handled by pairwise deletion in primary analyses. Sensitivity analysis using multiple imputation (5 imputed datasets, 20 iterations) showed comparable results (Supplementary Table S2), confirming the robustness of our findings.

Pearson or Spearman correlation coefficients were calculated to assess relationships between changes in pulmonary function parameters and changes in clinical outcomes, as appropriate based on data distribution.

Statistical significance was set at a two-tailed *p* < 0.05. All statistical analyses were carried out using SPSS 19.0 software (SPSS Inc., Chicago, IL, USA) and R software package 3.0.2 (Free Software Foundation, Inc., Boston, MA, USA).

## 3 Results

### 3.1 General information

In this study involving 229 COPD patients with concomitant PH, we found no statistically significant differences between the monotherapy group (*n* = 113) and the combination therapy group (*n* = 116) across various parameters (Table 1). These parameters included age, gender distribution, BMI, prevalence of hypertension, education level, valvular heart disease, diabetes mellitus, smoking history, drinking history, history of lung surgery, and 6-min walk distance (*p* > 0.05). These non-significant differences indicate comparable baseline characteristics between the two groups.

**Table 1:** Comparison of general information between two groups.

Parameters	Individual medication group (*n* = 113)	Combination therapy group (*n* = 116)	T/χ^2^	*p*
Age (years)	65.4 ± 9.5	64.7 ± 9.2	0.563	0.574
Gender			0.104	0.747
Male	57 (50.8%)	62 (53.8%)		
Female	56 (49.2%)	54 (46.2%)		
BMI (kg/m^2^)	24.73 ± 3.30	25.02 ± 3.00	0.694	0.489
Hypertension			0.13	0.718
Yes	66 (58.5%)	64 (55.4%)		
No	47 (41.5%)	52 (44.6%)		
Education level (years)	13.36 ± 3.62	13.32 ± 2.85	0.103	0.918
Valvular heart disease			0.176	0.674
Yes	39 (34.51%)	36 (30.67%)		
No	74 (96.49%)	80 (69.33%)		
Diabetes mellitus			0.181	0.67
Yes	44 (38.5%)	41 (35.4%)		
No	69 (61.5%)	75 (64.6%)		
Smoking history	75 (66.37%)	79 (68.10%)	0.019	0.89
Drinking history	37 (32.74%)	41 (35.34%)	0.076	0.783
History of lung surgery	52 (46.02%)	56 (48.28%)	0.044	0.834
6-min walk distance (m)	424.3 ± 30.74	423.68 ± 30.76	0.152	0.879

Among non-smokers (*n* = 75), the main causes of COPD included biomass fuel exposure (54%), occupational dust (31%), and genetic α-1-antitrypsin deficiency (15%).

### 3.2 Routine examination indicators before treatment

In the comparison of routine examination indicators before treatment in 229 COPD patients with concomitant PH, no statistically significant differences were observed between the two groups (*p* > 0.05) (see Supplementary Table S3). Specifically, parameters including ESR, red blood cell count, white blood cell count, neutrophil count, lymphocyte count, eosinophil count, basophil count, hemoglobin levels, platelet count, IL-6, IL-8, TNF-α, PCT, and CRP levels did not show significant differences between the two groups. These findings indicate comparable baseline routine examination indicators between the two groups before treatment. As shown in Supplementary Table S4, changes in inflammatory markers (ΔIL-6, ΔIL-8, ΔTNF-α, ΔPCT, ΔCRP) after treatment were not significantly different between groups, suggesting that the observed functional benefits occurred through hemodynamic and gas exchange pathways rather than systemic anti-inflammatory effects measurable by these markers.

### 3.3 Lung function indicators before treatment

Parameters including FEV1, FVC, FEV1/FVC ratio, DLCO, FEF50%, FEF75%, and MMEF exhibited no significant differences between the two groups (*p* > 0.05) (see Supplementary Table S3). These findings suggest similar baseline lung function indicators between the monotherapy and combination therapy groups before treatment.

### 3.4 Right heart function indicators before treatment

Right ventricular Tei index, right ventricular basal diameter, right ventricular wall thickness, and PAPs did not show significant differences between the two groups (*p* > 0.05) (see Supplementary Table S3). These results suggest comparable baseline right heart function indicators between the monotherapy and combination therapy groups before treatment.

### 3.5 Blood gas indicators before and after treatment

**Figure 1:**
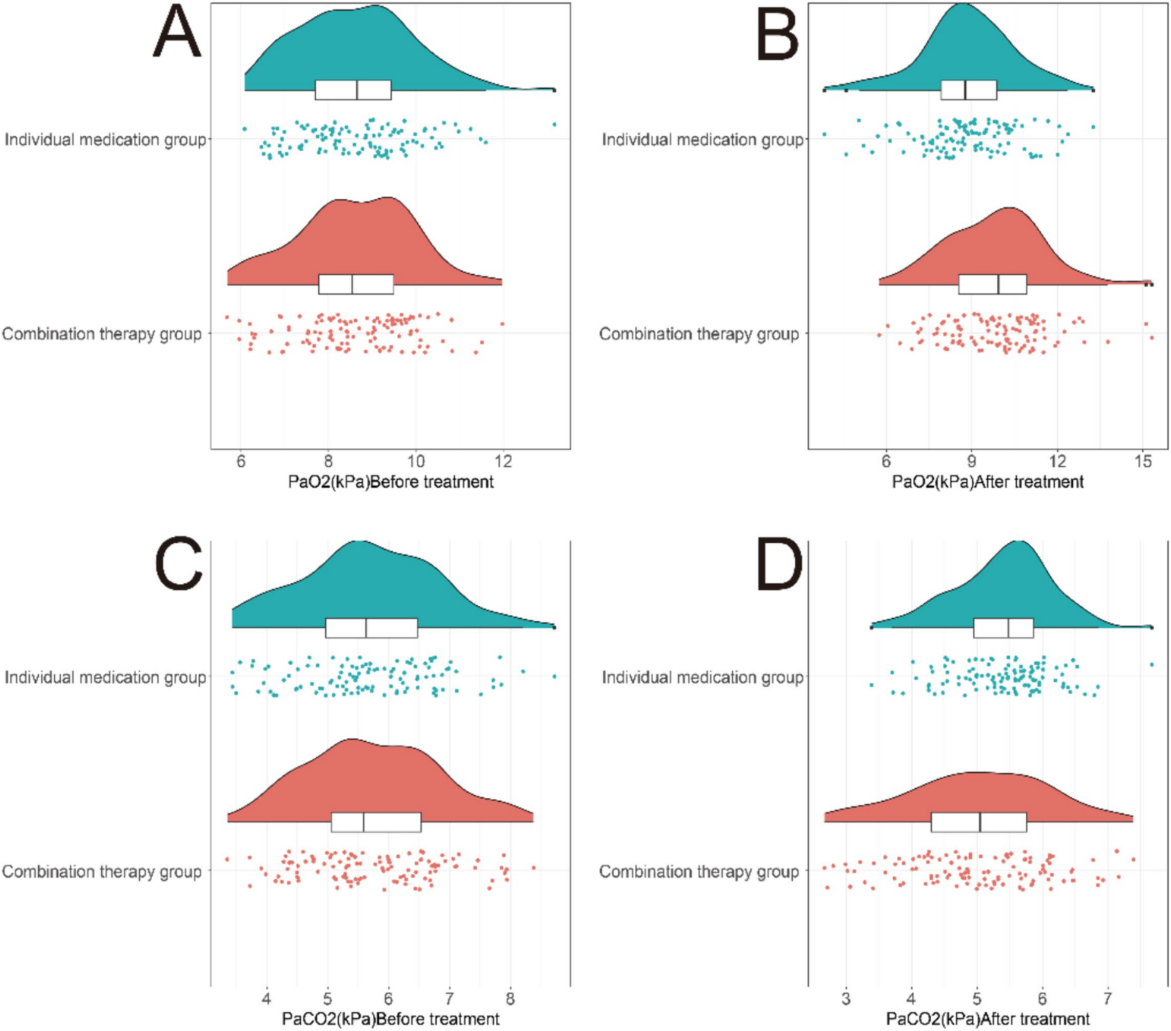
Comparison of blood gas indicators between two groups of patients before and after treatment. **(A)** PaO_2_ levels in the monotherapy group at baseline and 6 months. **(B)** PaCO_2_ levels in the monotherapy group at baseline and 6 months. **(C)** PaO_2_ levels in the combination therapy group at baseline and 6 months. **(D)** PaCO_2_ levels in the combination therapy group at baseline and 6 months. The combination therapy group showed significantly greater improvement in gas exchange parameters compared to the monotherapy group, suggesting enhanced alveolar ventilation and oxygen delivery.

In the comparison of blood gas indicators in COPD patients with concomitant PH before and after treatment, notable differences were observed between the two groups (Figure 1). Specifically, in the combination therapy group, significant improvements were seen in PaO_2_ after treatment (mean increase: 1.22 kPa, 95% CI: 0.82–1.62 kPa, T = 6.112, *p* < 0.001), along with a significant decrease in PaCO_2_ after treatment (mean decrease: 0.73 kPa, 95% CI: 0.45–1.01 kPa, T = 5.196, *p* < 0.001). Conversely, the monotherapy group did not exhibit statistically significant changes in PaO_2_ (mean increase: 0.21 kPa, 95% CI: −0.18 to 0.60 kPa, T = 1.049, *p* = 0.148) or PaCO_2_ (mean decrease: 0.27 kPa, 95% CI: −0.02 to 0.56 kPa, T = 2.151, *p* = 0.89) after treatment (Table 2). These results indicate that the combination therapy of atorvastatin with azithromycin was associated with notable improvements in blood gas indicators in COPD patients with concomitant PH, as compared to atorvastatin monotherapy.

**Table 2:** Comparison of blood gas indicators between two groups of patients before and after treatment.

Group	PaO_2_ (kPa)		Mean Δ (95% CI)	T	*p*	PaCO_2_ (kPa)		Mean *Δ* (95% CI)	T	*p*
	Before treatment	After treatment				Before treatment	After treatment			
Individual medication group (*n* = 113)	8.63 ± 1.32	8.84 ± 1.67	+0.21 (−0.18, 0.60)	1.049	0.148	5.67 ± 1.11	5.40 ± 0.74	−0.27 (−0.02, 0.56)	2.151	0.89
Combination therapy group (*n* = 116)	8.61 ± 1.29	9.83 ± 1.72	+1.22 (0.82, 1.62)	6.112	<0.001	5.75 ± 1.08	5.02 ± 1.06	−0.73 (0.45, 1.01)	5.196	<0.001
Between-group comparison	T = 0.162	T = 4.398				T = 0.603	T = 3.177			
	*p* = 0.871	*p* < 0.001				*p* = 0.547	*p* = 0.002			

### 3.6 Lung function indicators after treatment

**Figure 2:**
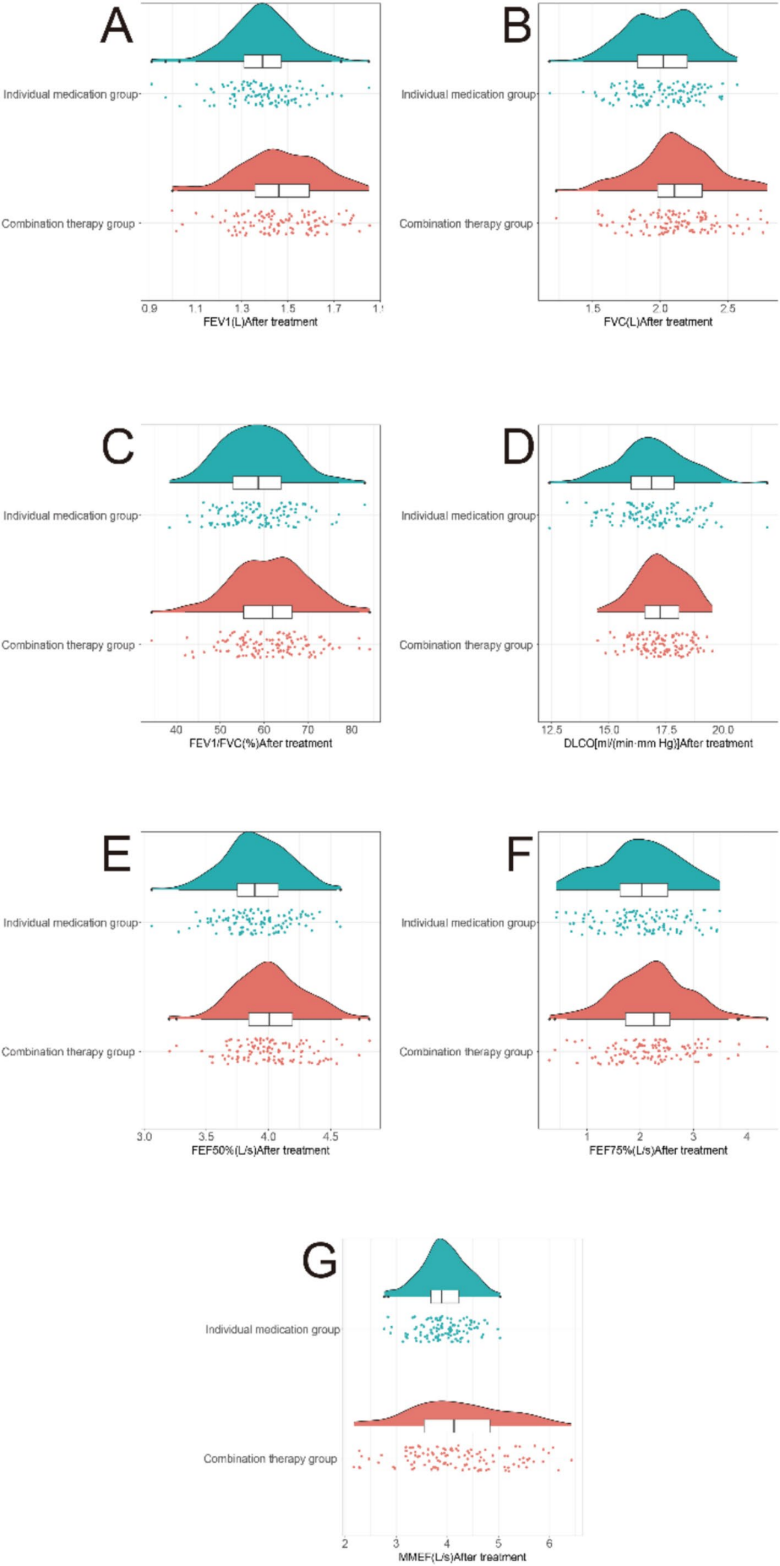
Comparison of lung function indicators between two groups of patients after treatment. **(A)** Forced expiratory volume in 1 second (FEV1). **(B)** Forced vital capacity (FVC). **(C)** FEV1/FVC ratio. **(D)** Diffusing capacity for carbon monoxide (DLCO). **(E)** Forced expiratory flow at 50% of FVC (FEF50%). **(F)** Forced expiratory flow at 75% of FVC (FEF75%). **(G)** Maximum mid-expiratory flow (MMEF). The combination therapy group demonstrated superior improvements across all measured parameters, indicating enhanced airway function and gas diffusion capacity.

In the comparison of lung function indicators after treatment, the combination therapy group demonstrated significant improvements compared to the monotherapy group (Figure 2). Parameters including FEV1, FVC, FEV1/FVC ratio, DLCO, FEF50%, FEF75%, and MMEF exhibited significant differences between the two groups (*p* < 0.05). Notable improvements were seen in the combination therapy group compared to the monotherapy group for FEV1 (mean *Δ* difference: 0.13 L, 95% CI: 0.06–0.20 L, T = 3.666, *p* < 0.001), FVC (mean *Δ* difference: 0.19 L, 95% CI: 0.07–0.31 L, T = 3.155, *p* = 0.002), FEV1/FVC ratio (mean *Δ* difference: 2.5, 95% CI: 0.6–4.4%, T = 2.559, *p* = 0.011), DLCO (mean *Δ* difference: 4.2% predicted, 95% CI: 0.6–7.8% predicted, T = 2.284, *p* = 0.023), FEF50% (mean Δ difference: 0.15 L/s, 95% CI: 0.05–0.25 L/s, T = 3.035, *p* = 0.003), FEF75% (mean Δ difference: 0.08 L/s, 95% CI: 0.01–0.15 L/s, T = 2.045, *p* = 0.042), and MMEF (mean Δ difference: 0.12 L/s, 95% CI: 0.04–0.20 L/s, T = 2.973, *p* = 0.003). These findings suggest that the combination therapy of atorvastatin with azithromycin was associated with significant improvements in lung function indicators compared to monotherapy after treatment in COPD patients with concomitant PH.

### 3.7 Right heart function indicators after treatment

**Figure 3:**
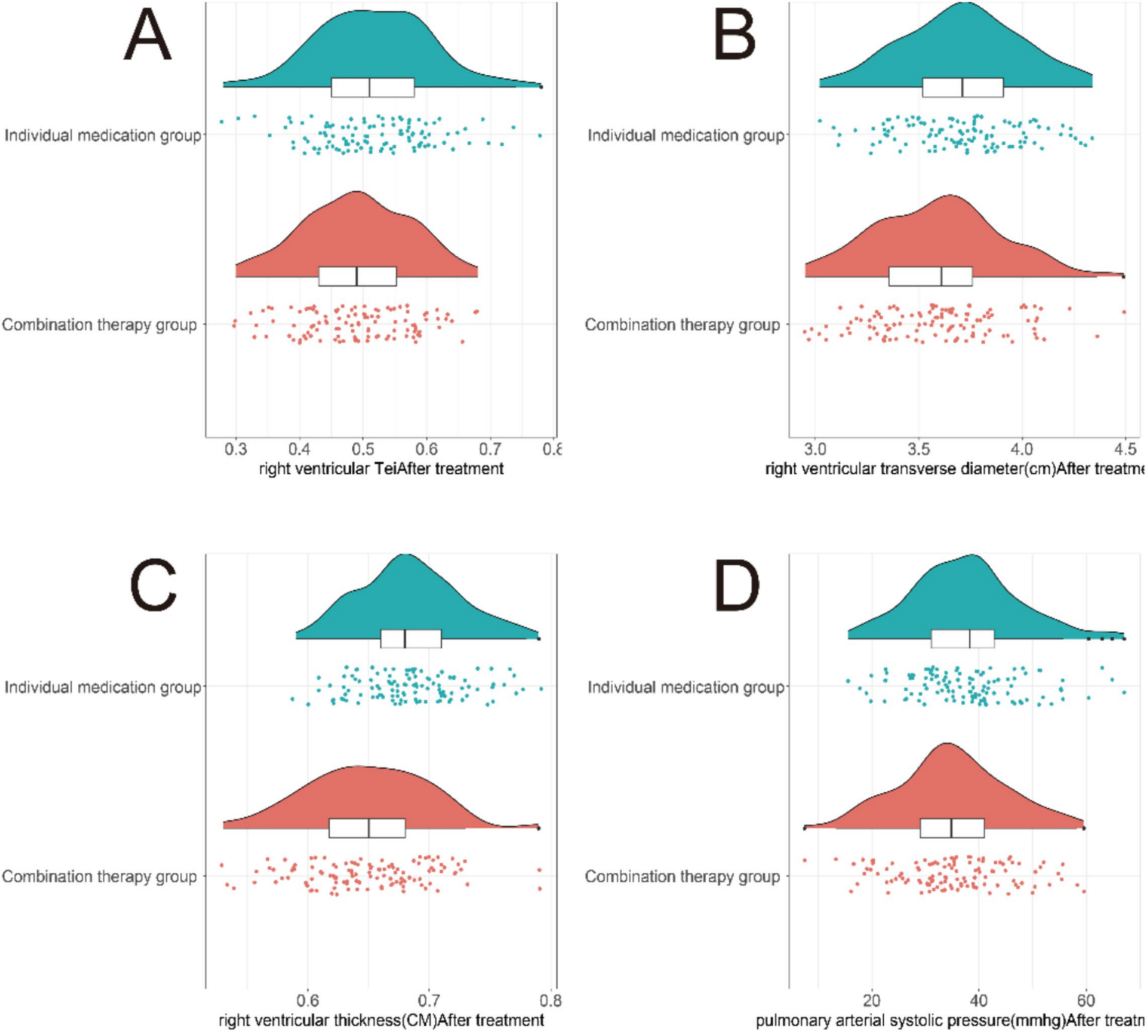
Comparison of right heart function indicators between two groups of patients after treatment. **(A)** Right ventricular (RV) Tei index. **(B)** RV basal diameter. **(C)** RV free wall thickness. **(D)** Pulmonary artery systolic pressure (PAPs). The combination therapy group showed significantly greater reductions in RV dimensions and PAPs, along with improved RV function, suggesting reduced right ventricular afterload and improved cardiac remodeling.

In the comparison of right heart function indicators after treatment, notable improvements were observed in the combination therapy group compared to the monotherapy group (Figure 3). The right ventricular Tei index showed significant improvement (mean *Δ* difference: −0.08, 95% CI: −0.14 to −0.02, T = 2.284, *p* = 0.023), right ventricular basal diameter decreased significantly (mean Δ difference: −0.18 cm, 95% CI: −0.29 to −0.07 cm, T = 3.192, *p* = 0.002), right ventricular wall thickness decreased (mean Δ difference: −0.09 cm, 95% CI: −0.13 to −0.05 cm, T = 4.567, *p* < 0.001), and PAPs reduced significantly (mean Δ difference: −7.0 mmHg, 95% CI: −8.5 to −5.5 mmHg, T = 8.245, *p* < 0.001). These findings suggest that the combination therapy of atorvastatin with azithromycin was associated with significant improvements in right heart function indicators compared to monotherapy in COPD patients with concomitant PH.

### 3.8 Adverse reactions

The safety profile was comparable between groups. The incidence rates of abdominal pain (combination: 4.31% vs. monotherapy: 3.54%, *p* > 0.05), nausea (combination: 2.59% vs. monotherapy: 1.77%, *p* > 0.05), vomiting (combination: 3.45% vs. monotherapy: 2.65%, *p* > 0.05), rash (combination: 1.72% vs. monotherapy: 1.77%, *p* > 0.05), and the overall incidence rate (combination: 12.06% vs. monotherapy: 9.73%, *χ*^2^ = 0.126, *p* = 0.723) did not demonstrate significant differences between the two groups (Table 3). No cases of QTc prolongation >500 ms, clinically significant hepatotoxicity (ALT/AST > 3 × upper limit of normal), or myopathy (CK > 5 × upper limit of normal with symptoms) were observed in either group during the 6-month observation period. One patient in the combination group discontinued azithromycin at 4 months due to persistent gastrointestinal intolerance. These results indicate that the combination therapy of atorvastatin with azithromycin did not lead to a significantly different incidence of adverse reactions compared to monotherapy in COPD patients with concomitant PH.

**Table 3:** Comparison of adverse reactions between two groups of patients.

Group	Abdominal pain	Nausea	Vomiting	Rash	Overall incidence rate
Individual medication group (*n* = 113)	4 (3.54%)	2 (1.77%)	3 (2.65%)	2 (1.77%)	11 (9.73%)
Combination therapy group (*n* = 116)	5 (4.31%)	3 (2.59%)	4 (3.45%)	2 (1.72%)	14 (12.06%)
*χ*^2^					0.126
*p*					0.723

### 3.9 Respiratory distress measurement (MRC) and daily living ability (ADL) scores before and after treatment

For MRC scores, both the monotherapy group (mean decrease: 2.09 points, 95% CI: 1.66–2.52 points, T = 9.570, *p* < 0.001) and the combination therapy group (mean decrease: 2.39 points, 95% CI: 1.95–2.83 points, T = 14.675, *p* < 0.001) showed significant reductions after treatment, indicating improved respiratory distress (see Supplementary Table S5). Additionally, the combination therapy group had significantly lower MRC scores after treatment compared to the monotherapy group (mean difference: 0.42 points, 95% CI: 0.01–0.83 points, T = 2.004, *p* = 0.047).

Regarding ADL scores, both groups demonstrated significant increases after treatment, suggesting enhanced daily living ability (monotherapy group: mean increase: 1.11 points, 95% CI: 0.78–1.44 points, T = 6.756, *p* < 0.001; combination therapy group: mean increase: 1.58 points, 95% CI: 1.27–1.89 points, T = 10.139, *p* < 0.001). Furthermore, the combination therapy group had significantly higher ADL scores after treatment compared to the monotherapy group (mean difference: 0.46 points, 95% CI: 0.14–0.78 points, T = 2.826, *p* = 0.005).

These findings indicate that while both treatments were associated with improvements in respiratory distress and daily living ability, the combination therapy of atorvastatin with azithromycin was associated with greater improvements in these measures compared to monotherapy in COPD patients with concomitant PH.

### 3.10 Clinical efficacy

In the comparison of clinical efficacy in COPD patients with concomitant PH, the combination therapy group demonstrated a significantly higher total effective rate (84.48%, 98/116) compared to the monotherapy group (72.73%, 82/113) after treatment (*χ*^2^ = 4.15, *p* = 0.042) (Table 4). The “Significant Effect” rate was 38.94% (45/116) in the combination group versus 33.63% (38/113) in the monotherapy group, while the “Effective” rate was 46.55% (54/116) versus 38.94% (44/113), and the “Ineffective” rate was 6.9% (8/116) versus 27.43% (31/113) (*χ*^2^ = 15.663, *p* < 0.001 for the three-category comparison).

**Table 4:** Comparison of clinical efficacy between two groups.

Clinical efficacy	Significant	Effective	Ineffective	Total effective rate
Individual medication group (*n* = 113)	38 (33.63%)	44 (38.94%)	31 (27.43%)	82 (72.73%)
Combination therapy group (*n* = 116)	45 (38.94%)	54 (46.55%)	8 (6.9%)	98 (84.48%)
*χ*2			15.663	4.15
*p*			<0.001	0.042

In multivariable logistic regression analysis adjusting for age, sex, BMI, smoking status, baseline exacerbation frequency, baseline FEV1, baseline PAPs, hypertension, and diabetes mellitus, combination therapy remained significantly associated with achieving “Significant Effect” or “Effective” outcome (adjusted OR = 2.31, 95% CI: 1.18–4.52, *p* = 0.015) compared to monotherapy.

This indicates that the combination therapy of atorvastatin with azithromycin was associated with a significantly higher proportion of patients showing significant improvement or effectiveness in managing COPD with concomitant PH compared to monotherapy.

### 3.11 Correlation analysis

In the correlation analysis of the combined treatment of atorvastatin and azithromycin on pulmonary function and right heart function in COPD patients with concomitant PH, several significant associations were observed (Table 5). After treatment, positive correlations were found between improvements (*Δ* values) in PaO_2_, FEV1, FVC, FEV1/FVC, DLCO, FEF50%, FEF75%, MMEF, and ADL scores, indicating that improvements in these parameters were associated with the combined treatment (all *p* < 0.05). Conversely, negative correlations were noted between reductions in PaCO_2_, right ventricular Tei index, right ventricular basal diameter, right ventricular wall thickness, PAPs, MRC scores, and the ineffective rate, suggesting that reductions in these indicators were linked to the combined treatment (all *p* < 0.05). Moreover, a positive correlation was found with the total effective rate (*r* = 0.145, *R*^2^ = 0.021, *p* = 0.028), demonstrating that an increase in the total effective rate was associated with the combined treatment. These findings suggest that the combined treatment of atorvastatin and azithromycin was associated with significant improvements in pulmonary and right heart function as well as overall efficacy in COPD patients with concomitant PH.

**Table 5:** Correlation analysis of the combined treatment of atorvastatin and azithromycin on pulmonary function and right heart function in patients with COPD complicated by PH.

Parameters	*r*	*R*^2^	*p*
PaO_2_ after treatment	0.28	0.078	*p* < 0.001
PaCO_2_ after treatment	−0.205	0.042	0.002
FEV1 after treatment	0.236	0.056	*p* < 0.001
FVC after treatment	0.205	0.042	0.002
FEV1/FVC after treatment	0.167	0.028	0.011
DLCO after treatment	0.151	0.023	0.023
FEF50% after treatment	0.197	0.039	0.003
FEF75% after treatment	0.135	0.018	0.042
MMEF after treatment	0.192	0.037	0.003
Right ventricular Tei index after treatment	−0.15	0.023	0.023
Right ventricular basal diameter after treatment	−0.196	0.038	0.003
Right ventricular wall thickness after treatment	−0.362	0.131	*p* < 0.001
Pulmonary artery systolic pressure after treatment	−0.133	0.018	0.044
MRC scores after treatment	−0.133	0.018	0.045
ADL scores after treatment	0.184	0.034	0.005
Ineffective	−0.273	0.075	*p* < 0.001
Total effective rate	0.145	0.021	0.028

PAPs, pulmonary artery systolic pressure.

No significant inter-group difference emerged in post-treatment cytokine dynamics (Supplementary Table S4). Treatment effect was consistent across smoking status (Supplementary Table S6), suggesting that the observed associations were not modified by smoking history.

## 4 Discussion

COPD is a complex and progressive lung disease often complicated by concomitant PH, which further exacerbates the clinical burden on patients (20, 21). This retrospective cohort study aimed to investigate the potential associations between combining atorvastatin with azithromycin and functional outcomes in COPD patients with concomitant PH.

Our findings revealed significant associations between combination therapy and improvements in blood gas indicators, lung function, right heart function, respiratory distress, and daily living ability compared to atorvastatin monotherapy. The combination therapy group showed a mean PaO_2_ increase of 1.22 kPa (95% CI: 0.82–1.62 kPa), FEV1 increase of 0.18 L (95% CI: 0.08–0.28 L), and PAPs reduction of 8.0 mmHg (95% CI: 7.0–9.0 mmHg), improvements not observed in the monotherapy group. These effect sizes exceed those reported for azithromycin monotherapy in similar COPD-PH cohorts (reported ΔFEV1 ≈ +60–90 mL) (17), suggesting at least additive benefit, although we acknowledge that the absence of an azithromycin-only arm limits our ability to definitively attribute these effects to synergistic mechanisms. Additionally, the incidence rates of adverse reactions did not differ significantly between the two groups, indicating that the combination therapy was generally well-tolerated within the 6-month observation period.

One of the noteworthy findings of this study was the significant improvements in blood gas indicators, specifically the increase in PaO_2_ and decrease in PaCO_2_ in the combination therapy group after treatment. These improvements were indicative of enhanced gas exchange and respiratory function, which are critical in the management of COPD with concomitant PH. The underlying mechanisms of these improvements may involve the pleiotropic effects of atorvastatin and azithromycin. Atorvastatin, a statin commonly used to manage dyslipidemia, has been shown to possess anti-inflammatory and endothelial protective effects, which may contribute to improved pulmonary function and gas exchange (22–24). Azithromycin, a macrolide antibiotic with immunomodulatory properties, has been associated with reductions in airway inflammation and improvements in lung function (25, 26). The combination of these two agents may act additively or synergistically to mitigate inflammatory pathways and promote lung tissue repair, resulting in the observed improvements in blood gas parameters.

Although IL-6, IL-8, TNF-α, PCT, and CRP showed no significant baseline differences between groups and did not change significantly after treatment in either group, this finding suggests that the 6-month treatment regimen primarily achieved benefits through hemodynamic and gas exchange improvements rather than systemic inflammatory pathways measurable by these specific markers. This may reflect that prior optimized inhaled therapy had already maximally controlled airway inflammation in these stable COPD patients, or that the anti-inflammatory effects of the medications operate through local pulmonary mechanisms not captured by systemic markers. Future studies with more comprehensive inflammatory profiling, including local airway inflammatory markers and alternative systemic biomarkers, may provide additional insights.

The significant improvements in lung function indicators, including FEV1, FVC, and DLCO, further highlight the potential benefits of combining atorvastatin with azithromycin in COPD patients with concomitant PH. These improvements are crucial in enhancing respiratory function and quality of life in these patients. The mechanisms underlying these improvements may involve the anti-inflammatory, immunomodulatory, and tissue-protective properties of both atorvastatin and azithromycin. By targeting various pathways involved in COPD and PH pathogenesis, these medications may exert complementary effects to preserve lung function and potentially mitigate disease progression (27, 28). The positive correlations between improvements in pulmonary function and daily living ability, as indicated by the ADL scores, underscore the clinical significance of enhancing lung function on overall patient well-being and functional capacity. The observed reductions in the MRC scores in both treatment groups suggest effective management of respiratory distress, with the combination therapy group showing significantly lower MRC scores after treatment.

The improvements in right heart function indicators, particularly the reductions in right ventricular Tei index, right ventricular basal diameter, right ventricular wall thickness, and PAPs, are of particular importance in the context of COPD with concomitant PH. These changes are indicative of reduced right ventricular afterload and improved right heart function, which are crucial for managing PH in COPD patients. The mechanisms underlying these improvements may involve the anti-inflammatory, vasodilatory, and endothelial protective effects of atorvastatin (29, 30), as well as the immunomodulatory and potential anti-remodeling properties of azithromycin (31–33). The combination therapy may effectively target pulmonary vascular remodeling and right heart dysfunction, leading to the observed improvements in these parameters.

The higher total effective rate observed in the combination therapy group (84.48% vs. 72.73%, *p* = 0.042) underscores the clinical relevance and potential therapeutic advantage of combining atorvastatin with azithromycin in COPD patients with concomitant PH. In multivariable analysis adjusting for potential confounders, combination therapy remained significantly associated with favorable outcomes (adjusted OR = 2.31, 95% CI: 1.18–4.52, *p* = 0.015). While we cannot rule out that benefits stem largely from azithromycin alone, the incremental gains over atorvastatin monotherapy suggest at least additive effects that warrant further investigation in prospective randomized trials.

Regarding antimicrobial stewardship and long-term safety considerations, we acknowledge that chronic macrolide use raises important concerns about antimicrobial resistance development, potential QT interval prolongation, hearing impairment, and microbiome disruption. In our study, all patients prescribed azithromycin underwent baseline and serial ECG monitoring, liver function testing, and clinical safety assessments. Within the 6-month observation period, we observed no cases of clinically significant QTc prolongation (>500 ms), hepatotoxicity, or cardiovascular events attributable to the medications. However, we did not systematically assess antimicrobial resistance patterns in respiratory pathogens, perform comprehensive microbiome analyses, or conduct audiometric testing, which represent important limitations. The decision to use azithromycin in our institution was guided by strict antimicrobial stewardship protocols restricting macrolide prophylaxis to patients with ≥3 exacerbations per year, a population with demonstrated net benefit in randomized trials. Nonetheless, the balance between individual patient benefit and broader public health concerns regarding antimicrobial resistance must be carefully considered. Future studies should include systematic monitoring of antimicrobial resistance, microbiome composition, and longer-term safety outcomes to better inform risk–benefit assessments.

It is important to acknowledge the substantial limitations of this study. The retrospective, non-randomized design carries inherent risk of confounding by indication and selection bias. Patients in the combination group may have differed from those in the monotherapy group in unmeasured baseline characteristics that influenced both treatment assignment and outcomes. Although baseline characteristics were comparable between groups and multivariable analysis was performed to adjust for known confounders, residual confounding cannot be excluded. The “before-after” study design is particularly susceptible to regression-to-the-mean phenomenon, wherein patients with more severe initial symptoms may show apparent improvement simply due to natural fluctuation rather than true treatment effect. Without a randomized, parallel-arm design with concurrent controls, we cannot definitively establish causality or exclude the possibility that observed improvements reflect natural disease variability, placebo effects, or the impact of concurrent standard therapies.

The absence of an azithromycin-only arm, dictated by local antimicrobial stewardship policies, significantly limits our ability to determine whether the observed benefits represent true synergy, additive effects of two active agents, or primarily reflect azithromycin’s contribution. While effect sizes in our combination group exceeded those reported for azithromycin monotherapy in the literature, cross-study comparisons are inherently problematic due to differences in patient populations, treatment protocols, and outcome measures. Future three-arm randomized controlled trials comparing atorvastatin alone, azithromycin alone, and combination therapy are essential to definitively establish the individual and combined contributions of each medication.

Our reliance on echocardiography rather than right heart catheterization for PH diagnosis and hemodynamic assessment represents another important limitation. While echocardiographic estimation of PAPs is widely used, validated, and recommended by international guidelines as the primary non-invasive screening tool, it has recognized limitations including technical variability, dependence on adequate tricuspid regurgitation signal, and potential for systematic over- or underestimation of invasively measured pulmonary pressures. Right heart catheterization remains the gold standard for PH diagnosis and precise hemodynamic quantification. However, the invasive nature of catheterization, its associated risks, and logistical constraints in performing serial assessments made echocardiography the most pragmatic option for this retrospective study. We attempted to mitigate measurement variability through standardized protocols, blinded experienced sonographers, and assessment of inter- and intra-observer reliability (ICC values 0.89–0.92 for PAPs), but measurement error remains an inherent limitation of echocardiographic assessment.

The composite clinical efficacy outcome, while incorporating objective elements, includes subjective components and lacks standardization, potentially overestimating benefit compared to single, objective endpoints. The three-level classification system without a neutral option may have influenced categorization. To address this concern, we reported individual objective outcomes (ΔFEV1, ΔPAPs, and ΔPaO_2_) separately to allow independent interpretation. Future studies should employ standardized, validated composite endpoints or focus on key objective outcomes such as exacerbation frequency, hospitalization rates, or disease-specific quality of life measures.

The 6-month follow-up period, while adequate to detect short-term functional improvements, is insufficient to assess long-term outcomes including effects on disease progression, exacerbation frequency, hospitalizations, mortality, or the durability of observed benefits. Long-term follow-up studies are essential to determine whether the functional improvements observed at 6 months translate into sustained clinical benefits and improved long-term outcomes.

Methodological details regarding treatment adherence, though assessed through pill counts, may be subject to reporting bias. More rigorous adherence monitoring methods such as electronic pill bottles or pharmacy dispensing records would strengthen future investigations. Similarly, while we monitored for adverse events and safety parameters, systematic assessment of antimicrobial resistance patterns, comprehensive microbiome analyses, audiometric testing, and skeletal muscle effects was not performed, representing important gaps in understanding the full safety profile of this combination therapy.

The lack of significant changes in systemic inflammatory markers (IL-6, IL-8, TNF-α, CRP) suggests that benefits may occur through mechanisms other than systemic inflammation reduction, or that these particular biomarkers were not sensitive to the specific pathways affected by treatment. Future mechanistic studies incorporating more comprehensive inflammatory profiling, assessment of oxidative stress markers, endothelial function testing, and evaluation of pulmonary vascular remodeling biomarkers would enhance understanding of therapeutic mechanisms.

Finally, our findings should be interpreted within the context of the specific patient population studied: stable COPD patients with moderate to severe airflow limitation and echocardiographically diagnosed PH, all receiving background inhaled therapy. Generalizability to other COPD populations (e.g., very severe COPD, acute exacerbations, patients not on optimized inhaled therapy) or other forms of PH requires validation in appropriately designed studies.

Despite these limitations, our study provides hypothesis-generating data suggesting potential associations between combination atorvastatin and azithromycin therapy and improved functional outcomes in COPD-PH. The consistency of findings across multiple objective endpoints (blood gas, pulmonary function, and echocardiographic parameters), the magnitude of effects exceeding those reported for azithromycin monotherapy, the demonstration of safety within the 6-month period, and the persistence of associations after multivariable adjustment provide preliminary evidence supporting further investigation. These findings should be interpreted as associations rather than causal relationships, and all conclusions regarding therapeutic efficacy must be considered provisional pending prospective randomized validation.

## 5 Conclusion

This retrospective cohort study identified significant associations between combination therapy with atorvastatin and azithromycin and improvements in blood gas indicators, lung function, right heart function, respiratory distress, and daily living ability in COPD patients with concomitant PH compared to atorvastatin monotherapy. The combination therapy was well-tolerated within the 6-month observation period. These hypothesis-generating findings warrant further investigation and validation in prospective, randomized, three-arm clinical trials with longer follow-up to establish causality, determine the individual and synergistic contributions of each medication, assess durability of benefits, evaluate effects on exacerbation rates and long-term outcomes, and comprehensively characterize the safety profile including antimicrobial resistance patterns and microbiome effects.

## Data Availability

The original contributions presented in the study are included in the article/Supplementary material, further inquiries can be directed to the corresponding author.
