# Supplementary material for: The effect of atorvastatin combined with azithromycin on lung function and right heart function in COPD patients with concomitant pulmonary hypertension
Source: Front Med (Lausanne). 2026 Jan 29;12:1667773. doi: 10.3389/fmed.2025.1667773 (PMC12894270; doi:10.3389/fmed.2025.1667773)
Supplement: Supplementary file 1 [file Table_1.docx]

# Supplementary Tables

## Supplementary Table S4. Baseline Maintenance Inhaled Therapy

| Inhaled regimen | Individual medication (n=113) | Combination therapy (n=116) | P value |
| --- | --- | --- | --- |
| LAMA only | 12 (10.6 %) | 13 (11.2 %) | 0.87 |
| LABA only | 6 (5.3 %) | 6 (5.2 %) | 0.97 |
| ICS + LABA | 82 (72.6 %) | 84 (72.4 %) | 0.90 |
| LABA + LAMA (dual) | 10 (8.8 %) | 11 (9.5 %) | 0.93 |
| ICS + LABA + LAMA (triple) | 3 (2.7 %) | 2 (1.7 %) | 0.65 |
| PDE‑4 inhibitor add‑on | 0 (0 %) | 0 (0 %) | — |

## Supplementary Table S5. Sensitivity Analysis for Δ PAPs

| Analysis set | Δ PAPs (mm Hg) Mean ± SD | 95 % CI | P vs Mono |
| --- | --- | --- | --- |
| Mono group – Complete‑case | -1.0 ± 6.0 | -2.1 to +0.1 | — |
| Mono group – Multiple imputation | -1.2 ± 6.1 | -2.2 to -0.2 | — |
| Combo group – Complete‑case | -8.0 ± 5.0 | -9.0 to -7.0 | <0.001 |
| Combo group – Multiple imputation | -8.1 ± 5.2 | -9.1 to -7.1 | <0.001 |

## Supplementary Table S1. Baseline Clinical, Laboratory, Pulmonary Function and Echocardiographic Characteristics

| Variable | Individual medication (n=113) | Combination therapy (n=116) | P value |
| --- | --- | --- | --- |
| ESR (mm h⁻¹) | 25 ± 10 | 24 ± 9 | 0.54 |
| RBC (10¹² L⁻¹) | 4.60 ± 0.50 | 4.65 ± 0.55 | 0.48 |
| WBC (10⁹ L⁻¹) | 7.2 ± 1.1 | 7.3 ± 1.2 | 0.62 |
| Neutrophils (%) | 60 ± 6 | 61 ± 7 | 0.40 |
| Lymphocytes (%) | 27 ± 5 | 26 ± 5 | 0.45 |
| Eosinophils (%) | 3.2 ± 0.8 | 3.1 ± 0.9 | 0.59 |
| Basophils (%) | 0.6 ± 0.2 | 0.6 ± 0.2 | 0.88 |
| Hemoglobin (g L⁻¹) | 135 ± 15 | 136 ± 14 | 0.67 |
| Platelets (10⁹ L⁻¹) | 255 ± 48 | 258 ± 50 | 0.71 |
| IL‑6 (pg mL⁻¹) | 22 ± 5 | 23 ± 6 | 0.56 |
| IL‑8 (pg mL⁻¹) | 19 ± 4 | 19 ± 4 | 0.80 |
| TNF‑α (pg mL⁻¹) | 26 ± 6 | 27 ± 7 | 0.49 |
| Procalcitonin (ng mL⁻¹) | 0.10 ± 0.04 | 0.11 ± 0.05 | 0.55 |
| CRP (mg L⁻¹) | 6.8 ± 2.0 | 6.9 ± 2.1 | 0.70 |
| FEV₁ (L) | 1.05 ± 0.25 | 1.07 ± 0.24 | 0.44 |
| FVC (L) | 2.20 ± 0.40 | 2.22 ± 0.39 | 0.53 |
| FEV₁/FVC (%) | 48.0 ± 5.0 | 47.5 ± 5.2 | 0.41 |
| DL_CO (% pred) | 55 ± 8 | 56 ± 8 | 0.60 |
| FEF50% (L s⁻¹) | 1.25 ± 0.33 | 1.27 ± 0.32 | 0.74 |
| FEF75% (L s⁻¹) | 0.48 ± 0.15 | 0.49 ± 0.16 | 0.68 |
| MMEF (L s⁻¹) | 0.62 ± 0.18 | 0.64 ± 0.17 | 0.72 |
| RV Tei index | 0.62 ± 0.08 | 0.63 ± 0.09 | 0.50 |
| RV basal diameter (cm) | 3.50 ± 0.30 | 3.52 ± 0.32 | 0.63 |
| RV wall thickness (cm) | 0.60 ± 0.10 | 0.61 ± 0.11 | 0.58 |
| PAPs (mm Hg) | 52 ± 6 | 53 ± 7 | 0.55 |

Supplementary Table S2. Cytokine Dynamics After 6‑Month Treatment

| Marker | Baseline Mono | 6 months Mono | Baseline Combo | 6 months Combo | Δ Change (Combo–Mono) | P_inter‑group |
| --- | --- | --- | --- | --- | --- | --- |
| IL‑6 (pg mL⁻¹) | 22 ± 5 | 21 ± 5 | 23 ± 6 | 22 ± 6 | 0 | 0.82 |
| IL‑8 (pg mL⁻¹) | 19 ± 4 | 18 ± 4 | 19 ± 4 | 18 ± 4 | 0 | 0.95 |
| TNF‑α (pg mL⁻¹) | 26 ± 6 | 25 ± 6 | 27 ± 7 | 26 ± 7 | 0 | 0.86 |
| Procalcitonin (ng mL⁻¹) | 0.10 ± 0.04 | 0.09 ± 0.04 | 0.11 ± 0.05 | 0.10 ± 0.05 | 0 | 0.78 |
| CRP (mg L⁻¹) | 6.8 ± 2.0 | 6.5 ± 2.1 | 6.9 ± 2.1 | 6.4 ± 2.0 | -0.2 | 0.71 |

## Supplementary Table S3. MRC and ADL Scores

| Scale | Time‑point | Individual medication | Combination therapy | Within‑group P | Between‑group P (post) |
| --- | --- | --- | --- | --- | --- |
| MRC score | Baseline | 5.88 ± 1.29 | 5.76 ± 1.35 | — |  |
| MRC score | 6 months | 3.79 ± 1.93 | 3.37 ± 1.12 | <0.001 | 0.047 |
| ADL score | Baseline | 3.90 ± 1.23 | 3.89 ± 1.12 | — |  |
| ADL score | 6 months | 5.01 ± 1.24 | 5.47 ± 1.25 | <0.001 | 0.005 |

## Supplementary Table S6. Treatment Effect Stratified by Smoking Status

| Outcome | Smoking status | Individual medication | Combination therapy | P_interaction |
| --- | --- | --- | --- | --- |
| Δ FEV₁ (L) | Current/Former | +0.05 ± 0.21 | +0.18 ± 0.26 | 0.88 |
| Δ FEV₁ (L) | Never‑smoker | +0.06 ± 0.19 | +0.17 ± 0.27 |  |
| Δ PAPs (mm Hg) | Current/Former | -1 ± 6 | -8 ± 5 | 0.92 |
| Δ PAPs (mm Hg) | Never‑smoker | -0.8 ± 6.3 | -7.8 ± 5.4 |  |
| Total effective rate (%) | Current/Former | 70.7 % | 83.5 % | 0.90 |
| Total effective rate (%) | Never‑smoker | 73.7 % | 86.5 % |  |
